# Potential association of certain microRNA gene polymorphisms with recurrent pregnancy loss susceptibility in Saudi women

**DOI:** 10.1371/journal.pone.0336432

**Published:** 2025-12-19

**Authors:** Aya Rabaa, Afrah Alkuriji, Aishah Kabbi, Samiah Almalki, Zainab Almasawi, Maha Daghestani, Hana Hakami, Jawaher Alzahrani

**Affiliations:** 1 Department of Zoology, College of Science, King Saud University, Riyadh, Saudi Arabia; 2 Department of Biology, College of Science, Qassim University, Qassim, Saudi Arabia; University of Vermont College of Medicine, UNITED STATES OF AMERICA

## Abstract

MicroRNA (miRNA) polymorphisms are increasingly recognized as important regulators of reproductive outcomes, but their role in recurrent pregnancy loss (RPL) is still unexplored in specific populations. This case control study investigated six miRNA polymorphisms (miR-10-A > T, miR-125-G > A, miR-146a-C > G, miR-149-T > C, miR-323b-C > T, miR-499-A > G) in 50 Saudi women with idiopathic (≥ 2 losses) and 50 matched controls (≥ 1 live birth, no loss history) using PCR Sanger sequencing. Significant associations were found for heterozygous genotypes of miR-146a-C > G (OR=2.29, 95% Cl:1.02–5.18, *p* = 0.046) and miR-149-T > C (OR=2.67, 95% Cl:1.08–6.61, *p* = 0.034) with higher prevalence in RPL patients versus controls, while other polymorphisms showed no significant association (*p* > 0.05). These results suggest miR-146a and miR-149 can contribute to RPL susceptibility in Saudi women, highlighting their potential as population-specific genetic biomarkers and underscoring the need for further research into miRNA-mediated pregnancy maintenance mechanisms.

## Introduction

Recurrent pregnancy loss, clinically termed as recurrent spontaneous abortion, poses significant emotional suffering for individuals enduring the condition, as it is considered one of the most complex pregnancy complications, affecting nearly 1–3% of couples trying to conceive [1]. Regardless of the differences in diagnostic criteria, RPL is most commonly described as two or more consecutive pregnancy losses prior to the 20 weeks of gestation. However, this is still a debatable definition, especially with respect to the criteria such as minimum threshold for the number of pregnancy losses, consecutiveness of losses and the cutoff for the gestational age [2]. To resolve this contention, WHO has recommended a guideline for defining the miscarriage as the loss of a pregnancy prior to the twenty second week bearing a fetal weight of less than five hundred grams [3].

The etiology of RPL is complex and multifactorial involving genetic abnormalities, anatomical malformation, endocrine dysfunction, placental pathology, infection, substance abuse (tobacco and alcohol), environmental factors, psychological stress and immunological disorders [4]. Among these, the genetic factors play significant role in RPL pathogenesis with numerous SNPs particularly microRNAs (miRNAs), being studied intensively in recent studies [5,6]. MiRNAs are small non-coding RNAs that function at the post-transcriptional level by binding to complementary sequences in the 3′-untranslated region of specific messenger RNAs, leading to mRNA degradation or inhibition of translation [7–9]. The microRNAs play significant roles in numerous pathological and physiological processes including cell division, differentiation, apoptosis, immune response and tissue remodeling. In addition to their broad influence, changes in the miRNA expression have been implicated in pregnancy-related complications such as preeclampsia, implantation failure and RPL [10–13].

According to many studies, approximately 30% of human genome is estimated to be regulated by miRNAs, underscoring their broad influence. Many miRNAs are highly expressed in reproductive tissues, suggesting their critical roles in maintaining successful pregnancies [5,14]. Beyond their role in reproduction, miRNA polymorphisms have emerged as important genetic modifiers in a wide spectrum of human diseases. In cancers, specific miRNA variants can alter the regulation of tumor suppressors and oncogenes, thereby affecting cell proliferation, apoptosis, and metastasis [15]. Similarly, in cardiovascular disease, miRNA polymorphisms have been linked to aberrant lipid metabolism, endothelial dysfunction, and vascular remodeling, while in diabetes, such variants influence insulin signaling pathways and β-cell survival, collectively highlighting their pleiotropic contribution to complex disorders including RPL [16,17].

Moreover, miRNA polymorphisms have also been associated with autoimmune diseases and reproductive pathologies [18]. Previous studies have associated SNPs in miRNAs like miR-149-rs2292832, miR-499-rs3746444, miR-10a-rs3809783, miR-323b-rs56103835, miR-125a-rs41275794/rs12976445 and miR-146a-rs2910164 with increased risk of RPL in different populations [19,20]. For instance, a SNP in pri-miR-10a has been shown to alter its maturation and potentially influence downstream gene regulation, contributing to RPL susceptibility in a Han-Chinese population [19]. Likewise, genetic variants in miR-146a and miR-196a2 have been linked to altered immune responses and inflammatory pathways in Iranian women with idiopathic RPL [20]. Notably, miR-146a has been involved in regulating the *Fas* gene, a critical mediator of oocyte apoptosis, suggesting a possible mechanistic link to RPL [21,22]. Based on these functional roles and previous genetic associations, this study investigated the association among six miRNA polymorphisms (miR-10-A > T, miR-125-G > A, miR-146a-C > G miR-149-T > G, miR-323b-C > T and miR-499-A > G) and idiopathic RPL in a cohort of Saudi women.

## Materials and methods

### Subjects selection

This study was conducted in accordance with the Declaration of Helsinki and approved by the Ethics Committee of King Saud University (22/0629/IRB). Written informed consent was obtained from all participants, including both patients and healthy controls, who voluntarily agreed to donate peripheral blood samples for genetic and biochemical analyses. Recruitment was carried out at the outpatient department of King Khaled University Hospital.

The study cohort comprised 50 women diagnosed with recurrent pregnancy loss (RPL), defined according to the American Society for Reproductive Medicine (ASRM) criteria as two or more consecutive clinical pregnancy losses before 20 weeks of gestation, confirmed by ultrasound or histopathological examination [23]. Inclusion criteria were women aged 20–45 years with a history of ≥2 idiopathic consecutive miscarriages. Exclusion criteria included uterine malformations (septate uterus, fibroids), parental chromosomal abnormalities, endocrine disorders (uncontrolled diabetes, thyroid dysfunction, hyperprolactinemia), autoimmune diseases (antiphospholipid syndrome, systemic lupus erythematosus), coagulation disorders, TORCH infections, exposure to teratogenic drugs, smoking or alcohol abuse.

The control group consisted of 50 age-matched women with at least one natural live birth, no history of miscarriage, regular menstrual cycles, and absence of infertility. The same exclusion criteria applied to the RPL group were also applied to the control group to eliminate potential confounding factors.

The anonymized demographic and clinical data of healthy controls and RPL patients are provided as Supporting Information (S1 File and S2 File). The demographic, clinical, and haematological characteristics of the study population are summarized in Table 1. No significant differences were observed between cases and controls with respect to age and body mass index (BMI), whereas the number of pregnancies differed significantly between the groups (p < 0.001).

**Table 1:** Demographic, clinical and hematological characteristics of population study.

Parameters	RPL group (n = 50)	Controls (n = 50)	P-value
Age (years)	36.08 ± 5.51 (24–44)	35.36 ± 5.10 (24–45)	0.58
BMI (kg/m²)	27.57 ± 4.23	27.95 ± 5.57	0.71
Number of pregnancies	4.98 ± 4.22 (3–32 losses)	3.40 ± 1.35 (2–7 live births)	<0.001

### DNA extraction and genotyping

About 6 mL blood sample was taken from each participant via venipuncture into EDTA containing tubes. DNA was extracted from the entire blood samples using the Puregene Blood Core Kit (Qiagen, Germany). DNA concentration and purity were assessed by a Nanodrop (NanoDrop® ND-2000) Spectrophotometer. Samples were selected for further examination with an A260/A280 ratio between 1.8 and 2.0.

### DNA sequencing and PCR amplification

PCR (polymerase chain reaction) was used to magnify the target regions containing single nucleotide polymorphisms (SNPs) in particular miRNAs. DNA samples were amplified using conventional polymerase chain reaction (PCR) using optimized thermal cycling parameters. Primer sequences and thermocycling conditions for each SNPs are given in Tables 2 and 3 respectively. A thermal cycler was used to perform PCR amplifications under controlled conditions. PCR amplicons were separated by agarose gel electrophoresis on 1.5% stained with SYBER Safe DNA Gel Stain and then visualized under UV transilluminator. Purified PCR amplicons were sequenced in the forward direction using Sanger sequencing on an ABI 3730xl DNA analyzer. The sequencing results were further visualized and analyzed using SnapGene software (GSL Biotech) to confirm the presence of complete nucleotide sequences and alignments. Representative sequencing chromatograms for SNP genotyping are provided in S1 Fig.

**Table 2:** Primer sequences used for amplification of selected miRNA polymorphisms.

Polymorphism	Forward Primer (5′ → 3′)	Reverse Primer (5′ → 3′)	Product size (bp)
miR-10 A > T	CCCAGAGCTCAAAACTAGAACA	CGTGGGGAGAGTTCAGGTA	387
miR-125 G > A/ T > C	CTGTTTTCCTTCCCTGTCTGTC	ACCTTTATGAAGTCACCACAGG	486
miR-146a C > G	CTGAACTCCTGGGCTCAAGA	AGCTACTTGGAACCCTGCTT	496
miR-149 T > C	CTGCCTTTGACTGCCGTG	GCCCGAAACACCCGTAAG	393
miR-323b C > T	CTGTGCACACTAATCCTGGC	GGCTGAGGATTAGGAGTGCT	387
miR-499 A > G	GAAGGCAGCATCGTTCCAG	GGAGCAGGGCCCTAAGTG	385

**Table 3:** PCR thermocycling conditions used for amplification of miRNA polymorphisms.

Step	Conditions
Initial denaturation	98 °C for 30 sec
Denaturation	95 °C for 30 sec
Annealing	63 °C for 30 sec
Extension	72 °C for 30 sec
Final extension	72 °C for 5 min

### Statistical analysis

Statistical analysis (Mean (±), standard deviation) was used to calculate continuous variables (age, body mass index, number of abortions and number of live births) using IBM SPSS software (version 20.0, IBM Corp., NY, USA). Chi-square (x^2^) tests were used to analyze the genotype and allele frequencies of the RPL and control groups. For association analysis, odds ratios (ORs) with 95% confidence intervals (CIs) and corresponding Z scores were calculated using the MedCalc statistical software (https://www.medcalc.org). Statistical significance was set at p > 0.05 for all analysis. To calculate the expected and observed genotype frequencies in the control group, Hardy-Weinberg Equilibrium (HWE) equation was used by chi-square (x^2^) test available on (https://www.had2know.org/academics/hardy-weinberg-equilibrium-calculator-2-alleles.html).

## Results

The genotype distribution of six mi RNA SNPs (miR-10 A > T, miR-125 G > A, miR-146a C > G, miR-149 T > C, miR-323b C > T, and miR-499 A > G) were examined in both RPL and control groups are displayed in Table 4. Representative sequencing chromatograms confirming SNP genotypes are shown in S1 Fig. Anonymized individual-level genotype and clinical data for all study participants are provided in the Supporting Information (S1 File and S2 File). Genotypes within the control groups were constant with Hardy-Weinberg Equilibrium (HWE) for miR-125 G > A, miR146a C > G, miR-323b C > T polymorphisms. and miR-499 A > G. However, miR-146a C > G showed a deviation from HWE in the control group.

**Table 4:** The genotype distribution of miR10 A > T, miR-125 G > A, miR125 T > C miR-146aC > G, miR-149T > C, miR-323b C > T, and miR-499 A > G Polymorphisms in the case and control groups.

Genotype	RPL(n = 50) (%)	Controls (n = 50) (%)	P-value	OR (95%CI)	z statistic
miR-146a C > G					
GG	16 (33.3)	24 (48)	0.1415	0.5417 (0.2392 to 1.2265)	1.47
GC	26 (54.2)	17 (34)	0.0459	2.2941 (1.0151 to 5.1845)	1.996
CC	6 (12.5)	9 (18)	0.4518	0.6508 (0.2125 to 1.9927)	0.752
HWE p-value	0.3587	0.0739			
miR-149 T > C					
CC	23 (48.9)	29 (63.04)	0.1722	0.5618 (0.2455 to 1.2857)	1.365
CT	20 (42.6)	10 (21.73)	0.0343	2.6667 (1.0751 to 6.6144)	2.116
TT	4 (8.5)	7 (15.21)	0.3227	0.5183 (0.1409 to 1.9068)	0.989
HWE p-value	0.9055	0.0030			
miR125 T > C					
CC	5 (10.4)	7 (14.58)	0.5388	0.6811 (0.2001 to 2.3180)	0.615
CT	19 (39.6)	17 (35.41)	0.6734	1.1947 (0.5223 to 2.7327)	0.421
TT	24 (50.0)	24 (50)	1.0000	1 (0.4493 to 2.2259)	0.000
HWE p-value	0.6713	0.1878			
miR-323b C > T					
CC	0 (0.0)	2 (4.081)	0.2849	0.1881 (0.0088 to 4.0208)	1.069
CT	16 (32.0)	22 (44.89)	0.1887	0.5775 (0.2547 to 1.3094)	1.315
TT	34 (68.0)	25 (51.02)	0.0870	2.04 (0.9015 to 4.6161)	1.711
HWE p-value	0.178	0.2882			
miR-499 A > G					
AA	19 (43.2)	17 (37.77)	0.6037	1.2518 (0.5361 to 2.9227)	0.519
AG	18 (40.9)	20 (44.44)	0.7361	0.8654 (0.3733 to 2.0061)	0.337
GG	7 (15.9)	8 (17.77)	0.8139	0.875 (0.2878 to 2.6605)	0.235
HWE p-value	0.4413	0.6192			
miR10 A > T Monomorphic (no variation	N/A				

### Relationship between miRNA polymorphisms and RPL

No statistically significant difference (p > 0.05) in the genotype distribution were observed between RPL cases and controls for miR-125 G > A, miR-323b C > T and miR-499.A > G, indicating no evident correlation with RPL risk. However, a statistically significant association (p < 0.05) was identified for miR-146a C > G and miR-149 T > C polymorphisms:

#### miR-146a C > G rs2910164.

This SNP is intergenic variant with alleles C > G that is located on chromosome 5 (position 160485411) within the MIR146A gene. The RPL group had a considerably higher frequency of heterozygous CG genotype than in controls. The risk of RPL was significantly higher for carriers of the CG genotype: OR=2.29, 95% CL = 1.02–5.18, Z = 1.996, p = 0.0459. This suggest that rs2910164 CG genotype may serve as a genetic risk factor for RPL in this population.

#### miR-149a T > C rs22928332.

This SNP is categorized as an intronic variant and is found on chromosome 2 (position 240456086) in the MIR149 gene. Being more common among cases than controls, the TC genotype was significantly linked to RPL: OR=2.67, 95% CL = 1.08–6.61, Z = 2.116, p = 0.0343. This result indicates a potential role of miR-149 TC heterozygosity in the etiology of RPL.

#### miR-10A A > T monomorphism (rs3809783).

No genotype variation was detected for the miR-10A A > T (rs3809783) monomorphism. All members of both case and control groups were homozygous for allele A and indicating a monomorphic distribution. For this reason, statistical or HWE analysis was not performed for this SNP due to the absence of allelic variation.

## Discussion

MicroRNAs (miRNAs) are crucial post-transcriptional regulatory elements that play vital roles in complex biological processes, including centromere function, cell proliferation, stem cell differentiation and division, fat and cholesterol metabolism and most importantly female reproduction. They play most essential role in regulating oogenesis and spermatogenesis, as well as their precise control of embryo implantation and maternal-fetal communication [24–26]. Several studies have demonstrated the role of miRNAs in the female reproductive system across different mammalian species such as bovine, that have shown different expressions of miRNAs between small and large follicles or between healthy and atretic follicles. These miRNAs are involved in follicular cell proliferation, steroidogenesis, luteinization and oocyte maturation. Moreover, during the third day of the estrous cycle, miRNAs were also exhibited different expressions involved in the Wnt signaling, transforming growth factor (TGF-beta) signaling, axon guidance and apoptosis. By the day seven, the differentially expressed miRNAs shifted to the metabolism of vitamins, cofactors, lipids, lipoproteins amino acids such as cysteine and methionine [27,28].

Studies in humans and mice have also revealed low expression levels of the let-7b gene within granulosa cells of mature oocytes compared to the immature oocytes, suggesting the potential role in oocyte maturation [29,30]. Let-7b was first discovered in *Caenorhabditis elegans* and is highly conserved in humans and consists of 12 members of miRNA involved in development, stem cell biology, aging and metabolism [31]. miR-146a is one of the important regulatory molecules that play a role in controlling the expression of many genes particularly FOXL2, CCND2 and FAS [32]. FOXl2 (Forkhead box L2) belongs to FOX gene family, and highly conserved expression in adult human ovaries and play an effective role in ovarian and cellular differentiation [33,34]. FOXL2 also shows its highest levels of expression in the endometrium during the proliferative phase [35]. Its inactivation in murine models leads to infertility due to myometrial disorganization and vascular defects [36]. Similarly, CCND2 its predominantly expressed in granulosa cells and is essential for their proliferation during ovarian folliculogenesis [37]. It encodes cyclin D2 that facilitates in the transition from the G1 to the S phase during the cell cycle and its deficiency stops the cell cycle [20]. Moreover, miR-146a negatively regulates FAS expression by binding to its 3′-UTR which reduces apoptotic signaling [38]. FAS is reduced in women who are conflicting from recurrent pregnancy loss and unexplained infertility which results in the downregulation of apoptotic signaling in the epithelial cells during embryo implantation [39]. miR-146a itself expression is controlled by NF-κB signaling pathway and its upregulation enhances the survival and viability of mesenchymal stem cell (MSC) [40,41]. Suzuki et al. showed that treatment of MSCs with diazoxide (DZ) increased miR-146a expression and promoted cell survival via NF-κB pathway. Inhibition of NF-κB pathway or miR-146a knockdown mitigated this effect, indicating the critical function of miR-146a in cell survival [42]. Functionally, SNP rs2910164 within miR-146a may alter the stem-loop structure of its precursor, thereby reducing processing efficiency and mature miRNA abundance, which could dysregulate FOXL2, CCND2, and FAS expression, affecting folliculogenesis and endometrial receptivity [43].

miR149a is considered as a pro-apoptotic miRNA by inhibiting the expression of serine/threonine protein kinase AKT1 and transcription factor E2F1, both regulate cell proliferation and survival [44]. AKT1 is a multi-functional protein and involved in numerous cellular processes like growth and metabolism, while E2F1 regulates the G1/S phase transition and apoptosis [45]. SNP rs2292832 within miR-149 has been shown to influence Drosha and Dicer processing and strand selection, potentially altering its ability to downregulate AKT1 and E2F1. This dysregulation may disrupt the balance between granulosa cell proliferation and apoptosis, a key factor in successful embryo implantation [46]. miR-499 directly targets SOX6 gene which enhances the activity of terminal binding protein 2 (CtBP2), a repressor of transcription of fibroblast growth factor-3 (FGF-30). FGF-30 is essential for stimulating cell proliferation and differentiation, especially during the early stages of embryonic tissue development [47]. Similarly, miR-125 plays a critical role in organogenesis and adult tissues development, and associated with the TGF-β signaling which is vital for reproductive function and immune modulation. miR-125 also regulates angiogenesis in the endometrium and immune cell differentiation (T helper cells-Th17, T cells-Treg), and has been linked to abnormal pregnancy outcomes (preeclampsia when overexpressed) [48,49].

miR-10 family plays a pivotal role in repressing granulosa cell (GC) proliferation and induced apoptosis by targeting Brain-derived neurotrophic factor (BDNF), a key regulator of early follicular development and ovulation. Moreover, the miR-10 family and the TGF-β pathway form a negative feedback regulatory mechanism in granulosa cells [50]. Essential ovarian hormones and growth factors such as FSH, FGF9 and TGF-β pathway ligands (TGFβ1, Activin A, BMP4 and BMP15) inhibits miR-10a expression in GCs. Conversely, the miR-10 family suppresses multiple key genes within the TGF-β signaling pathway [51,52]. Furthermore, miR-323b negatively regulates paired-box 8 (Pax8), a transcription factor essential for embryo development, central nervous system, angiogenesis, immune regulation, and tumor metastasis as well as for the regulation of cell proliferation and differentiation [53,54]. However, patients with RPL show lower Pax8 expression and higher miR-323 levels [55]. These functional associations indicate that SNPs in miRNA genes may contribute to RPL pathogenesis by altering miRNA maturation, stability, or target binding, ultimately affecting key reproductive processes such as folliculogenesis, implantation, and immune modulation.

In our study, we investigated the association between certain SNPs in miRNA genes and prevalence of RPL in Saudi women, focusing on following variants miR-10-rs3809783, pri-miR-125a-rs41275794 and rs12976445, miR-146a-rs2910164, miR-149-rs2292832, miR-323b-rs56103835, miR-499A-rs3746444. Our results showed a significant positive relationship (p < 0.05) between susceptibility to RPL and two SNPs: miR-149T > C (rs2292832) and miR-146aC > G (rs2910164). For miR-146aC > G, our findings contrast with a meta-analysis of three studies which reported no statistically significant association with RPL [20,53,54]. However, we observed significantly higher genotype frequencies of this polymorphism in RPL cases compared to healthy controls (p < 0.05). Regarding miR-149T > C, our results align with a study conducted by reporting a significant association with RPL risk [56]. In contrast, they conflict with other reports that found no such association. For pri-miR-125a-rs12976445, miR-323b-rs56103835 and miR-499A-rs3746444, differential prevalence was observed between RPL cases and controls, but these differences were not statistically significant (p > 0.05). Previous studies and meta-analysis have reported that these SNPs may contribute to the risk of RPL [51,57,58], but our results did not support statistically significant association in Saudi population.

Possible reasons for observed deviation from Hardy–Weinberg equilibrium (HWE) in certain loci include small sample size, potential population stratification within studied cohort or technical errors in genotyping. However, all genotypes were rechecked to minimize chances of such errors. Additionally, unmeasured environmental exposures (smoking status, dietary habits, body mass index), lifestyle factors and other genetic variants may have acted as residual confounders, potentially influencing the observed associations.

This study has several important limitations. First, the small sample size may have reduced statistical power, particularly for detecting SNPs with small to modest associations. Second, deviation from Hardy–Weinberg equilibrium in some SNPs could reflect sampling bias or stratification. Third, the restriction to a Saudi population enhances population homogeneity but limits the generalizability of the findings to other ethnic groups. Fourth, the cross-sectional case–control design precludes causal inference. Finally, the absence of functional assays limits our ability to directly determine whether the identified SNPs alter miRNA maturation, stability, or target regulation. Future research should address these limitations by incorporating larger, multi-ethnic cohorts, collecting detailed environmental and lifestyle data, performing in vitro functional studies to elucidate the mechanistic role of these genetic variants.

Future studies should focus on multicenter cohorts with larger sample sizes to validate our findings across diverse populations. Moreover, functional studies are required to investigate the biological role of the identified miRNA SNPs in regulating gene expression and reproductive pathways. Integration of genomic, transcriptomic, and epigenetic approaches may further enhance our understanding of the molecular basis of recurrent pregnancy loss.

In conclusion, our study suggests that the miR-146aC > G and miR-149 T > C polymorphisms are significantly associated with increased risk of RPL in the Saudi population. These findings highlight the potential of miRNA related polymorphisms as biomarkers for reproductive outcomes and contribute to the increasing amount of data on genetic factors underlying RPL.

## Supporting information

S1 Fig Representative sequencing chromatograms of miRNA polymorphisms. Chromatograms illustrating single nucleotide polymorphisms (SNPs) detected in the study. miR-10 A > T: homozygous wild-type (A/A) in recurrent pregnancy loss (RPL) and control groups. miR-125 G > A: homozygous wild-type (G/G) in RPL and control groups. miR-125 T > C: homozygous (C/C) in RPL group, heterozygous (C/T) in RPL group, and homozygous wild-type (T/T) in control group. miR-146a C > G: homozygous (G/G) in RPL group, heterozygous (G/C) in RPL group, and homozygous wild-type (C/C) in control group. miR-149 T > C: homozygous (C/C) in RPL group, heterozygous (T/C) in RPL group, and homozygous wild-type (T/T) in control group. miR-223 C > T: homozygous (T/T) in RPL group, heterozygous (T/C) in RPL group, and homozygous wild-type (C/C) in control group. miR-499 A > G: homozygous (G/G) in RPL group, heterozygous (A/G) in RPL group, and homozygous wild-type (A/A) in control group. These chromatograms confirm the accuracy and reliability of SNP genotyping across cases and controls.
(TIF)

S1 File Anonymized dataset for healthy non-pregnant female participants. This file contains fully anonymized demographic and clinical variables used in the analysis, with no direct or indirect identifiers. All data included comply with participant consent and ethical requirements.
(PDF)

S2 File Anonymized dataset for females with recurrent pregnancy loss (RPL). This file includes anonymized demographic, clinical, and reproductive history data for non-pregnant female participants diagnosed with recurrent pregnancy loss (RPL). All information has been fully anonymized and complies with ethical and privacy requirements.
(PDF)

## References

[1] Rai R, Regan L. Recurrent miscarriage. Lancet. 2006;368(9535):601–11. doi: 10.1016/S0140-6736(06)69204-0 16905025

[2] Shaker M, Shalabi T, Gaber KR, Amr K. Association of miRNA-27a and leptin polymorphisms with recurrent pregnancy loss in Egyptian women. Meta Gene. 2020;24:100617. doi: 10.1016/j.mgene.2019.100617

[3] Dbstet A. WHO: recommended definitions, terminology and format for statistical tables related to the perinatal period and use of a new certificate for cause of perinatal deaths. Modifications recommended by FIGO as amended October 14, 1976. Acta Obstet Gynecol Scand. 1977;56(3):247–53. doi: 10.3109/00016347709162009 560099

[4] Choi YS, Kwon H, Kim JH, Shin JE, Choi Y, Yoon TK, et al. Haplotype-based association of ACE I/D, AT1R 1166A>C, and AGT M235T polymorphisms in renin-angiotensin-aldosterone system genes in Korean women with idiopathic recurrent spontaneous abortions. Eur J Obstet Gynecol Reprod Biol. 2011;158(2):225–8. doi: 10.1016/j.ejogrb.2011.04.028 21636204

[5] Ryan BM, Robles AI, Harris CC. Genetic variation in microRNA networks: the implications for cancer research. Nat Rev Cancer. 2010;10(6):389–402. doi: 10.1038/nrc2867 20495573 PMC2950312

[6] Al-Shorafa H, Sharif F. MicroRNA in a case of unexplained recurrent pregnancy loss. J Clin Case Rep. 2012;2(238):2.

[7] Larsen EC, Christiansen OB, Kolte AM, Macklon N. New insights into mechanisms behind miscarriage. BMC Med. 2013;11:154. doi: 10.1186/1741-7015-11-154 23803387 PMC3699442

[8] O’Toole AS, Miller S, Haines N, Zink MC, Serra MJ. Comprehensive thermodynamic analysis of 3’ double-nucleotide overhangs neighboring Watson-Crick terminal base pairs. Nucleic Acids Res. 2006;34(11):3338–44. doi: 10.1093/nar/gkl428 16820533 PMC1500867

[9] Wang C-Y, Wang S-G, Wang J-L, Zhou L-Y, Liu H-J, Wang Y-F. Effect of miRNA-27a and leptin polymorphisms on risk of recurrent spontaneous abortion. Med Sci Monit. 2016;22:3514–22. doi: 10.12659/msm.897147 27694792 PMC5049305

[10] Moore MS, Blobel G. The GTP-binding protein Ran/TC4 is required for protein import into the nucleus. Nature. 1993;365(6447):661–3. doi: 10.1038/365661a0 8413630

[11] Liu L, Liu Y, Min L, Zhou Z, He X, Xie Y, et al. Most Pleiotropic effects of gene knockouts are evolutionarily transient in yeasts. Mol Biol Evol. 2024;41(9):msae189. doi: 10.1093/molbev/msae189 39238468 PMC11414406

[12] Baek D, Villén J, Shin C, Camargo FD, Gygi SP, Bartel DP. The impact of microRNAs on protein output. Nature. 2008;455(7209):64–71. doi: 10.1038/nature07242 18668037 PMC2745094

[13] Sayed D, Abdellatif M. MicroRNAs in development and disease. Physiol Rev. 2011;91(3):827–87. doi: 10.1152/physrev.00006.2010 21742789

[14] Ardekani AM, Naeini MM. The role of MicroRNAs in human diseases. Avicenna J Med Biotechnol. 2010;2(4):161–79. 23407304 PMC3558168

[15] Hu Z, Chen J, Tian T, Zhou X, Gu H, Xu L, et al. Genetic variants of miRNA sequences and non-small cell lung cancer survival. J Clin Invest. 2008;118(7):2600–8. doi: 10.1172/JCI34934 18521189 PMC2402113

[16] Zhi H, Wang L, Ma G, Ye X, Yu X, Zhu Y, et al. Polymorphisms of miRNAs genes are associated with the risk and prognosis of coronary artery disease. Clin Res Cardiol. 2012;101(4):289–96. doi: 10.1007/s00392-011-0391-3 22159951

[17] Ciccacci C, Di Fusco D, Cacciotti L, Morganti R, D’Amato C, Greco C, et al. MicroRNA genetic variations: association with type 2 diabetes. Acta Diabetol. 2013;50(6):867–72. doi: 10.1007/s00592-013-0469-7 23532299

[18] Pankiewicz K, Laudański P, Issat T. The role of noncoding RNA in the pathophysiology and treatment of premature ovarian insufficiency. Int J Mol Sci. 2021;22(17):9336. doi: 10.3390/ijms22179336 34502244 PMC8430788

[19] Li Y, Wang X-Q, Zhang L, Lv X-D, Su X, Tian S, et al. A SNP in pri-miR-10a is associated with recurrent spontaneous abortion in a Han-Chinese population. Oncotarget. 2016;7(7):8208–22. doi: 10.18632/oncotarget.7002 26824181 PMC4884987

[20] Babakhanzadeh E, Danaei H, Abedinzadeh M, Ashrafzadeh HR, Ghasemi N. Association of miR-146a and miR196a2 genotype with susceptibility to idiopathic recurrent pregnancy loss in Iranian women: a case-control study. Int J Reprod Biomed. 2021;19(8):725–32. doi: 10.18502/ijrm.v19i8.9620 34568733 PMC8458919

[21] Panzan MQ, Mattar R, Maganhin CC, dos Simões RS, Rossi AGZ, da Motta ELA, et al. Evaluation of FAS and caspase-3 in the endometrial tissue of patients with idiopathic infertility and recurrent pregnancy loss. Eur J Obstet Gynecol Reprod Biol. 2013;167(1):47–52. doi: 10.1016/j.ejogrb.2012.10.021 23218678

[22] Wang X, Xing Y, Wang Y, Du Z, Zhang C, Gao J. Association of microRNA gene polymorphisms with recurrent spontaneous abortion: an updated meta‑analysis. Exp Ther Med. 2023;25(4):179. doi: 10.3892/etm.2023.11878 37006879 PMC10061040

[23] Practice Committee of the American Society for Reproductive Medicine. Evaluation and treatment of recurrent pregnancy loss: a committee opinion. Fertil Steril. 2012;98(5):1103–11. doi: 10.1016/j.fertnstert.2012.06.048 22835448

[24] Brennecke J, Hipfner DR, Stark A, Russell RB, Cohen SM. bantam encodes a developmentally regulated microRNA that controls cell proliferation and regulates the proapoptotic gene hid in Drosophila. Cell. 2003;113(1):25–36. doi: 10.1016/s0092-8674(03)00231-9 12679032

[25] Kotaja N. MicroRNAs and spermatogenesis. Fertil Steril. 2014;101(6):1552–62. doi: 10.1016/j.fertnstert.2014.04.025 24882619

[26] Salilew-Wondim D, Gebremedhn S, Hoelker M, Tholen E, Hailay T, Tesfaye D. The Role of MicroRNAs in Mammalian Fertility: From Gametogenesis to Embryo Implantation. Int J Mol Sci. 2020;21(2):585. doi: 10.3390/ijms21020585 31963271 PMC7014195

[27] Kim YJ, Ku S-Y, Kim YY, Liu HC, Chi SW, Kim SH, et al. MicroRNAs transfected into granulosa cells may regulate oocyte meiotic competence during in vitro maturation of mouse follicles. Hum Reprod. 2013;28(11):3050–61. doi: 10.1093/humrep/det338 23980055

[28] Sontakke SD, Mohammed BT, McNeilly AS, Donadeu FX. Characterization of microRNAs differentially expressed during bovine follicle development. Reproduction. 2014;148(3):271–83. doi: 10.1530/REP-14-0140 24920665

[29] Su J-L, Chen P-S, Johansson G, Kuo M-L. Function and regulation of let-7 family microRNAs. Microrna. 2012;1(1):34–9. doi: 10.2174/2211536611201010034 25048088

[30] Cho SH, An HJ, Kim KA, Ko JJ, Kim JH, Kim YR, et al. Single nucleotide polymorphisms at miR-146a/196a2 and their primary ovarian insufficiency-related target gene regulation in granulosa cells. PLoS One. 2017;12(8):e0183479. doi: 10.1371/journal.pone.0183479 28841705 PMC5571913

[31] Uhlenhaut NH, Treier M. Foxl2 function in ovarian development. Mol Genet Metab. 2006;88(3):225–34. doi: 10.1016/j.ymgme.2006.03.005 16647286

[32] Carlsson P, Mahlapuu M. Forkhead transcription factors: key players in development and metabolism. Dev Biol. 2002;250(1):1–23. doi: 10.1006/dbio.2002.0780 12297093

[33] Governini L, Carrarelli P, Rocha ALL, Leo VD, Luddi A, Arcuri F, et al. FOXL2 in human endometrium: hyperexpressed in endometriosis. Reprod Sci. 2014;21(10):1249–55. doi: 10.1177/1933719114522549 24520083

[34] Bellessort B, Bachelot A, Heude É, Alfama G, Fontaine A, Le Cardinal M, et al. Role of Foxl2 in uterine maturation and function. Hum Mol Genet. 2015;24(11):3092–103. doi: 10.1093/hmg/ddv061 25687138

[35] Han Y, Xia G, Tsang BK. Regulation of cyclin D2 expression and degradation by follicle-stimulating hormone during rat granulosa cell proliferation in vitro. Biol Reprod. 2013;88(3):57. doi: 10.1095/biolreprod.112.105106 23349233

[36] Bertoli C, Skotheim JM, de Bruin RAM. Control of cell cycle transcription during G1 and S phases. Nat Rev Mol Cell Biol. 2013;14(8):518–28. doi: 10.1038/nrm3629 23877564 PMC4569015

[37] Alexandrov PN, Dua P, Lukiw WJ. Up-regulation of miRNA-146a in progressive, age-related inflammatory neurodegenerative disorders of the human CNS. Front Neurol. 2014;5:181. doi: 10.3389/fneur.2014.00181 25324823 PMC4179622

[38] Suzuki Y, Kim HW, Ashraf M, Haider HK. Diazoxide potentiates mesenchymal stem cell survival via NF-kappaB-dependent miR-146a expression by targeting Fas. Am J Physiol Heart Circ Physiol. 2010;299(4):H1077-82. doi: 10.1152/ajpheart.00212.2010 20656888 PMC2957349

[39] Hatfield SD, Shcherbata HR, Fischer KA, Nakahara K, Carthew RW, Ruohola-Baker H. Stem cell division is regulated by the microRNA pathway. Nature. 2005;435(7044):974–8. doi: 10.1038/nature03816 15944714

[40] Lin R-J, Lin Y-C, Yu AL. miR-149* induces apoptosis by inhibiting Akt1 and E2F1 in human cancer cells. Mol Carcinog. 2010;49(8):719–27. doi: 10.1002/mc.20647 20623644

[41] Duggal S, Jailkhani N, Midha MK, Agrawal N, Rao KVS, Kumar A. Defining the Akt1 interactome and its role in regulating the cell cycle. Sci Rep. 2018;8(1):1303. doi: 10.1038/s41598-018-19689-0 29358593 PMC5778034

[42] O’Neill M, Ruthig VA, Haller M, Zhelyazkova B, White JT, Thirumavalavan N, et al. Developmental genetics of the male reproductive system. Human Reproductive and Prenatal Genetics. Elsevier; 2023. pp. 3–28. doi: 10.1016/b978-0-323-91380-5.00021-6

[43] Meshkat M, Tanha HM, Naeini MM, Ghaedi K, Sanati MH, Meshkat M, et al. Functional SNP in stem of mir-146a affects Her2 status and breast cancer survival. Cancer Biomark. 2016;17(2):213–22. doi: 10.3233/CBM-160633 27434289 PMC13020485

[44] Murakami A, Ishida S, Thurlow J, Revest JM, Dickson C. SOX6 binds CtBP2 to repress transcription from the Fgf-3 promoter. Nucleic Acids Res. 2001;29(16):3347–55. doi: 10.1093/nar/29.16.3347 11504872 PMC55854

[45] Guo S, Lu J, Schlanger R, Zhang H, Wang JY, Fox MC, et al. MicroRNA miR-125a controls hematopoietic stem cell number. Proc Natl Acad Sci U S A. 2010;107(32):14229–34. doi: 10.1073/pnas.0913574107 20616003 PMC2922532

[46] Xu L, Zhou X, Qiu M-T, Yin R, Wu Y-Q, Xu L. Lack of association between hsa-miR-149 rs2292832 polymorphism and cancer risk: a meta-analysis of 12 studies. PLoS One. 2013;8(9):e73762. doi: 10.1371/journal.pone.0073762 24040059 PMC3764043

[47] Leotta M, Biamonte L, Raimondi L, Ronchetti D, Di Martino MT, Botta C, et al. A p53-dependent tumor suppressor network is induced by selective miR-125a-5p inhibition in multiple myeloma cells. J Cell Physiol. 2014;229(12):2106–16. doi: 10.1002/jcp.24669 24819167

[48] Sharif K, Sharif Y, Watad A, Yavne Y, Lichtbroun B, Bragazzi NL, et al. Vitamin D, autoimmunity and recurrent pregnancy loss: More than an association. Am J Reprod Immunol. 2018;80(3):e12991. doi: 10.1111/aji.12991 29923244

[49] Lee E, Jeong YI, Park SM, Lee JY, Kim JH, Park SW, et al. Beneficial effects of brain-derived neurotropic factor on in vitro maturation of porcine oocytes. Reproduction. 2007;134(3):405–14. doi: 10.1530/REP-06-0288 17709559

[50] Dominguez MA, Cho N, Zhang B, Neal MS, Foster WG. Brain-derived neurotrophic factor expression in granulosa lutein cells. Reprod Biomed Online. 2011;22(1):17–24. doi: 10.1016/j.rbmo.2010.09.001 21115268

[51] Wang X-Q, Li Y, Su X, Zhang L, Liu C-M, Liu H, et al. Haplotype-based association of two SNPs in miR-323b with unexplained recurrent spontaneous abortion in a Chinese Han population. J Cell Physiol. 2018;233(8):6001–17. doi: 10.1002/jcp.26415 29271476

[52] Arakawa T, Fukuda S, Hirata T, Neriishi K, Wang Y, Takeuchi A, et al. PAX8: a highly sensitive marker for the glands in extragenital endometriosis. Reprod Sci. 2020;27(8):1580–6. doi: 10.1007/s43032-020-00186-7 32430717

[53] Jeon YJ, Choi YS, Rah H, Kim SY, Choi DH, Cha SH, et al. Association study of microRNA polymorphisms with risk of idiopathic recurrent spontaneous abortion in Korean women. Gene. 2012;494(2):168–73. doi: 10.1016/j.gene.2011.12.026 22222140

[54] Parveen F, Agrawal S. Recurrent miscarriage and micro-RNA among north Indian women. Reprod Sci. 2015;22(4):410–5. doi: 10.1177/1933719114529376 24700052 PMC4812686

[55] Hu S, Gan H, Yang F. Significance analysis of PAX8 expression in endometrial carcinoma. Medicine (Baltimore). 2022;101(42):e31159. doi: 10.1097/MD.0000000000031159 36281161 PMC9592497

[56] Alipour M, Abtin M, Hosseinzadeh A, Maleki M. Association between miR-146a C> G, miR-149 T> C, miR-196a2 T> C, and miR-499 A> G polymorphisms and susceptibility to idiopathic recurrent pregnancy loss. J Assist Reprod Genet. 2019;36:2237–44. 31605260 10.1007/s10815-019-01573-z PMC6885461

[57] Manzoor U, Pandith AA, Amin I, Wani S, Sanadhya D, Lone TA, et al. Implications of decreased expression of miR-125a with respect to its variant allele in the pathogenesis of recurrent pregnancy loss: a study in a high incidence zone. J Clin Med. 2022;11(13):3834. doi: 10.3390/jcm11133834 35807118 PMC9267497

[58] Bahari G, Taheri M, Mokhtari M, Moudi M, Majidpour M, Ghadimi HS. Association between Mir-499, Mir-27a, and Mir-146a polymorphisms and their susceptibility to recurrent spontaneous abortion; in silico analysis. Turkish J Obst Gynecol. 2024;21(3):158. 10.4274/tjod.galenos.2024.74419 PMC11589323 39228203

