# Supplementary figures and images for: Potential association of certain microRNA gene polymorphisms with recurrent pregnancy loss susceptibility in Saudi women
Source: PLoS One. 2025 Dec 19;20(12):e0336432. doi: 10.1371/journal.pone.0336432 (PMC12716758; doi:10.1371/journal.pone.0336432)

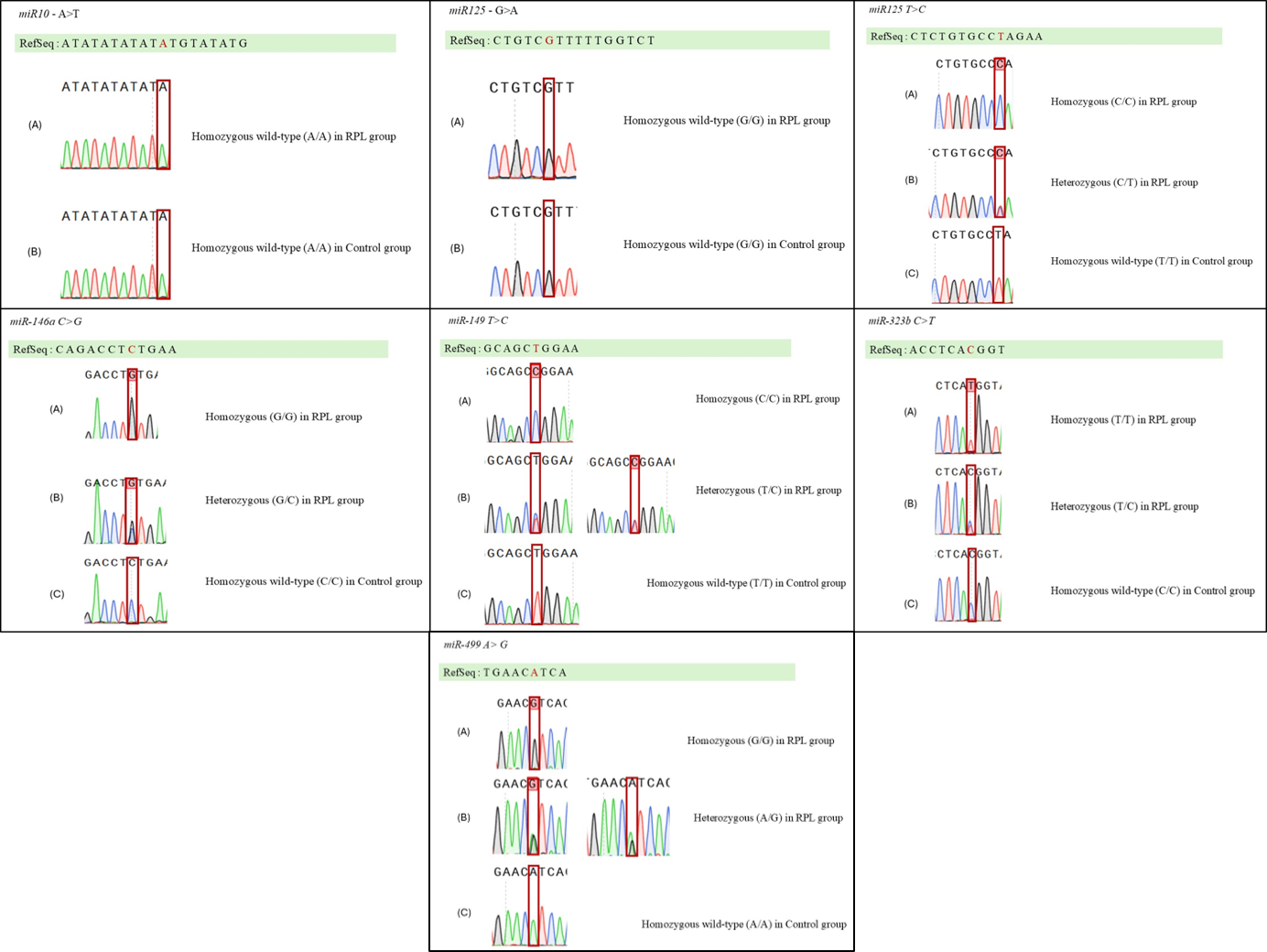

Supplement: S1 Fig — Chromatograms illustrating single nucleotide polymorphisms (SNPs) detected in the study. miR-10 A > T: homozygous wild-type (A/A) in recurrent pregnancy loss (RPL) and control groups. miR-125 G > A: homozygous wild-type (G/G) in RPL and control groups. miR-125 T > C: homozygous (C/C) in RPL group, heterozygous (C/T) in RPL group, and homozygous wild-type (T/T) in control group. miR-146a C > G: homozygous (G/G) in RPL group, heterozygous (G/C) in RPL group, and homozygous wild-type (C/C) in control group. miR-149 T > C: homozygous (C/C) in RPL group, heterozygous (T/C) in RPL group, and homozygous wild-type (T/T) in control group. miR-223 C > T: homozygous (T/T) in RPL group, heterozygous (T/C) in RPL group, and homozygous wild-type (C/C) in control group. miR-499 A > G: homozygous (G/G) in RPL group, heterozygous (A/G) in RPL group, and homozygous wild-type (A/A) in control group. These chromatograms confirm the accuracy and reliability of SNP genotyping across cases and controls. (TIF) [file pone.0336432.s001.tif]
