# Supplementary material for: Potential association of certain microRNA gene polymorphisms with recurrent pregnancy loss susceptibility in Saudi women
Source: PLoS One. 2025 Dec 19;20(12):e0336432. doi: 10.1371/journal.pone.0336432 (PMC12716758; doi:10.1371/journal.pone.0336432)
Supplement: S1 File — This file contains fully anonymized demographic and clinical variables used in the analysis, with no direct or indirect identifiers. All data included comply with participant consent and ethical requirements. (PDF) [file pone.0336432.s002.pdf]

IDs	Age (years)	Weight (kg)	Height (m)	BMI	Consanguinity	Children	Birth Type	Employment	Abortion
C01	30–39	58–62	1.57–1.61	23–24	No	2	Cesarean	Yes	0
C02	30–39	48–52	1.48–1.52	22–23	No	4	Natural	Yes	0
C03	30–39	81–85	1.58–1.62	32–33	Yes (cousin)	3	Natural	No	0
C04	20–29	55–59	1.63–1.67	20–21	No	2	Natural	No	0
C05	30–39	70–74	1.57–1.61	28–29	No	3	Natural	Yes	0
C06	30–39	88–92	1.58–1.62	35–36	No	3	Natural	Yes	0
C07	30–39	69–73	1.53–1.57	29–30	Yes (cousin)	3	Natural	No	0
C08	40–49	74–78	1.61–1.65	28–29	No	5	Natural	Yes	0
C09	40–49	92–96	1.55–1.59	38–39	No	3	Cesarean	Yes	0
C10	30–39	96–100	1.61–1.65	36–37	No	4	Natural	No	0
C11	40–49	73–77	1.58–1.62	29–30	No	2	Natural	Yes	0
C12	30–39	53–57	1.63–1.67	20–21	No	2	Natural	No	0
C13	40–49	96–100	1.63–1.67	36–37	No	2	Natural	No	0
C14	30–39	83–87	1.62–1.66	31–32	Yes (cousin)	6	Natural	No	0
C15	20–29	51–55	1.54–1.58	21–22	No	2	Natural	No	0
C16	20–29	78–82	1.58–1.62	31–32	No	2	Cesarean	No	0
C17	20–29	47–51	1.57–1.61	19–20	No	2	Natural- Cesarean	No	0
C18	30–39	73–77	1.56–1.60	30–31	No	2	Cesarean	No	0
C19	30–39	58–62	1.58–1.62	23–24	No	3	Natural	Yes	0
C20	30–39	75–79	1.52–1.56	32–33	No	6	Natural- Cesarean	Yes	0
C21	30–39	74–78	1.65–1.69	27–28	No	3	Natural	No	0
C22	30–39	56–60	1.54–1.58	23–24	No	2	Natural	No	0
C23	30–39	52–56	1.54–1.58	22–23	No	2	Natural	No	0
C24	20–29	47–51	1.46–1.50	22–23	Yes (cousin)	2	Natural	No	0
C25	30–39	53–57	1.51–1.55	23–24	Yes (cousin)	3	Natural	Yes	0
C26	30–39	67–71	1.52–1.56	29–30	No	4	Natural- Cesarean	No	0
C27	30–39	69–73	1.50–1.54	30–31	Yes (cousin)	3	Natural	No	0

C28	30–39	51–55	1.50–1.54	22–23	No	3	Natural	Yes	0
C29	40–49	108–112	1.51–1.55	47–48	No	6	Natural	No	0
C30	40–49	70–74	1.55–1.59	29–30	No	5	Natural- Cesarean	Yes	0
C31	30–39	67–71	1.56–1.60	27–28	No	4	Natural	No	0
C32	20–29	56–60	1.58–1.62	22–23	No	2	Natural	Yes	0
C33	40–49	58–62	1.54–1.58	24–25	No	4	Natural	Yes	0
C34	30–39	57–61	1.58–1.62	23–24	No	4	Natural	No	0
C35	30–39	79–83	1.55–1.59	32–33	Yes (cousin)	4	Natural	Yes	0
C36	30–39	53–57	1.48–1.52	24–25	Yes (cousin)	5	Natural- Cesarean	Yes	0
C37	30–39	74–78	1.53–1.57	31–32	No	3	Natural- Cesarean	No	0
C38	30–39	83–87	1.60–1.64	32–33	Yes (cousin)	2	Cesarean	No	0
C39	30–39	93–97	1.57–1.61	37–38	Yes (cousin)	3	Natural	Yes	0
C40	40–49	51–55	1.46–1.50	24–25	No	4	Natural	No	0
C41	30–39	58–62	1.48–1.52	26–27	Yes (cousin)	4	Natural	No	0
C42	30–39	74–78	1.54–1.58	31–32	Yes (cousin)	6	Natural	No	0
C43	30–39	76–80	1.60–1.64	29–30	No	2	Natural	Yes	0
C44	30–39	67–71	1.53–1.57	28–29	No	5	Natural	No	0
C45	20–29	56–60	1.58–1.62	22–23	No	2	Natural	No	0
C46	30–39	60–64	1.53–1.57	25–26	Yes (cousin)	3	Natural	Yes	0
C47	40–49	48–52	1.52–1.56	21–22	No	5	Natural	No	0
C48	30–39	70–74	1.55–1.59	29–30	No	4	Cesarean	Yes	0
C49	30–39	55–59	1.54–1.58	23–24	No	7	Natural	No	0
C50	30–39	66–70	1.53–1.57	28–29	No	3	Cesarean	Yes	0
