# Supplementary material for: Potential association of certain microRNA gene polymorphisms with recurrent pregnancy loss susceptibility in Saudi women
Source: PLoS One. 2025 Dec 19;20(12):e0336432. doi: 10.1371/journal.pone.0336432 (PMC12716758; doi:10.1371/journal.pone.0336432)
Supplement: S2 File — This file includes anonymized demographic, clinical, and reproductive history data for non-pregnant female participants diagnosed with recurrent pregnancy loss (RPL). All information has been fully anonymized and complies with ethical and privacy requirements. (PDF) [file pone.0336432.s003.pdf]

IDs	Age (years)	Weight (kg)	Height (m)	BMI	Consanguinity	Children	Birth Type	Employment	Abortion
P01	31–35	76–85	1.66–1.75	30–31	No	2	Natural	No	4
P02	41–45	56–65	1.46–1.55	26–27	No	4	Natural	Yes	4
P03	36–40	36–45	1.46–1.55	18–19	Yes (cousin)	3	Natural	Yes	6
P04	41–45	56–65	1.56–1.65	22–23	No	6	Natural	No	5
P05	31–35	66–75	1.66–1.75	25–26	No	2	Cesarean	Yes	3
P06	26–30	66–75	1.46–1.55	27–28	No	1	Natural	Yes	5
P07	36–40	66–75	1.46–1.55	31–32	No	0		Yes	4
P08	41–45	66–75	1.56–1.65	27–28	No	3	Natural	Yes	6
P09	31–35	56–65	1.46–1.55	24–25	No	1	Natural	No	11
P10	21–25	56–65	1.46–1.55	26–27	No	0		Yes	5
P11	21–25	36–45	1.46–1.55	17–18	Yes (cousin)	0		No	4
P12	41–45	66–75	1.56–1.65	26–27	No	0		No	8
P13	36–40	76–85	1.56–1.65	31–32	No	2	Natural- Cesarean	Yes	6
P14	31–35	66–75	1.56–1.65	30–31	Yes (cousin)	3	Cesarean	No	5
P15	36–40	76–85	1.66–1.75	26–27	Yes (cousin)	0		No	32
P16	31–35	66–75	1.56–1.65	29–30	No	0		No	5
P17	41–45	46–55	1.56–1.65	21–22	No	3	Natural	No	6
P18	41–45	76–85	1.56–1.65	28–29	No	1	Natural	Yes	5
P19	31–35	56–65	1.46–1.55	25–26	No	2	Natural	Yes	3
P20	36–40	76–85	1.56–1.65	30–31	Yes (cousin)	5	Natural	No	4
P21	36–40	56–65	1.66–1.75	22–23	Yes (cousin)	3	Cesarean	No	4
P22	26–30	46–55	1.56–1.65	20–21	Yes (cousin)	0		No	3
P23	21–25	56–65	1.46–1.55	25–26	No	0		No	3
P24	26–30	66–75	1.56–1.65	28–29	Yes (cousin)	2	Natural- Cesarean	No	5
P25	36–40	56–65	1.46–1.55	30–31	Yes (cousin)	1	Cesarean	Yes	6
P26	36–40	86–95	1.56–1.65	32–33	No	4	Natural	No	3
P27	21–25	56–65	1.46–1.55	25–26	Yes (cousin)	1	Natural	No	3
P28	31–35	66–75	1.56–1.65	28–29	No	2	Natural	Yes	3

P29	41–45	96–105	1.76–1.85	27–28	Yes (cousin)	5	Natural	No	3
P30	36–40	66–75	1.56–1.65	31–32	Yes (cousin)	1	Cesarean	Yes	3
P31	36–40	76–85	1.46–1.55	32–33	Nothing	3	Natural	No	3
P32	36–40	66–75	1.56–1.65	25–26	No	5	Natural	No	4
P33	36–40	56–65	1.46–1.55	26–27	Yes (cousin)	5	Natural- Cesarean	No	3
P34	41–45	66–75	1.56–1.65	27–28	Yes (cousin)	1	Natural	No	3
P35	31–35	66–75	1.66–1.75	25–26	No	0		No	6
P36	41–45	76–85	1.56–1.65	29–30	No	0		Yes	3
P37	31–35	66–75	1.56–1.65	28–29	Yes (cousin)	2	Natural- Cesarean	No	3
P38	36–40	96–105	1.56–1.65	38–39	No	2	Cesarean	Yes	7
P39	31–35	56–65	1.46–1.55	28–29	No	2	Natural- Cesarean	No	4
P40	41–45	66–75	1.66–1.75	24–25	No	4	Natural- Cesarean	Yes	3
P41	41–45	86–95	1.56–1.65	32–33	No	1	Cesarean	Yes	6
P42	36–40	56–65	1.56–1.65	26–27	No	4	Natural	No	3
P43	36–40	46–55	1.56–1.65	22–23	No	0		Yes	7
P44	36–40	76–85	1.56–1.65	33–34	No	1	Cesarean	No	5
P45	36–40	76–85	1.56–1.65	34–35	Yes (cousin)	6	Natural	No	5
P46	26–30	76–85	1.46–1.55	35–36	Yes (cousin)	1	Natural	No	3
P47	31–35	56–65	1.66–1.75	22–23	No	0		No	4
P48	26–30	56–65	1.56–1.65	23–24	No	2	Natural	No	3
P49	41–45	76–85	1.66–1.75	27–28	No	3	Natural- Cesarean	Yes	4
P50	31–35	66–75	1.56–1.65	27–28	Yes (cousin)	3	Cesarean	Yes	3
